# Stage 1 Registered Report: Refinement of tickling protocols to improve positive animal welfare in laboratory rats

**DOI:** 10.12688/f1000research.125649.2

**Published:** 2022-11-18

**Authors:** Vincent Bombail, Sarah M. Brown, Jessica E. Martin, Simone L. Meddle, Michael Mendl, Emma S.J. Robinson, Tayla J. Hammond, Birte L. Nielsen, Megan R. LaFollette, Ignacio Vinuela-Fernandez, Emma K.L. Tivey, Alistair B. Lawrence

**Affiliations:** 1 Animal Behaviour and Welfare group, Scotland’s Rural College (SRUC), Edinburgh, EH9 3RG, UK; 2 The Roslin Institute and Royal (Dick) School of Veterinary Studies, University of Edinburgh, Edinburgh, EH25 9RG, UK; 3 Bristol Veterinary School, University of Bristol, Bristol, BS40 5DU, UK; 4 School of Physiology, Pharmacology and Neuroscience, University of Bristol, Bristol, BS8 1TD, UK; 5 Universities Federation for Animal Welfare, Wheathampstead, AL4 8AN, UK; 6 The North American 3Rs Collaborative, Denver, CO, 80202, USA; 7 Bioresearch Veterinary Services, University of Edinburgh, Edinburgh, EH16 4SB, UK

**Keywords:** rat tickling, playful handling, protocol, positive animal welfare, affective neuroscience, laboratory animal welfare

## Abstract

Rat tickling is a heterospecific interaction for experimenters to mimic the interactions of rat play, where they produce 50 kHz ultrasonic vocalisations (USV), symptoms of positive affect; tickling can improve laboratory rat welfare. The standard rat tickling protocol involves gently pinning the rat in a supine position. However, individual response to this protocol varies. This suggests there is a risk that some rats may perceive tickling as only a neutral experience, while others as a positive one, depending on how tickling is performed. Based on our research experiences of the standard tickling protocol we have developed a playful handling (PH) protocol, with reduced emphasis on pinning, intended to mimic more closely the dynamic nature of play.

We will test whether our PH protocol gives rise to more uniform increases in positive affect across individuals relative to protocols involving pinning. We will compare the response of juvenile male and female Wistar rats as: Control (hand remains still against the side of the test arena), P0 (PH with no pinning), P1 (PH with one pin), P4 (PH with four pins). P1 and P4 consist of a background of PH, with treatments involving administration of an increasing dosage of pinning per PH session.

We hypothesise that rats exposed to handling protocols that maximise playful interactions (where pinning number per session decreases) will show an overall increase in total 50 kHz USV as an indicator of positive affect, with less variability. We will explore whether behavioural and physiological changes associated with alterations in PH experience are less variable.

We propose that maximising the numbers of rats experiencing tickling as a positive experience will reduce the variation in response variables affected by tickling and increase the repeatability of research where tickling is applied either as a social enrichment or as a treatment.

Research highlights **Scientific benefits**

Heterospecific play with rats can be used to study the physiology of positive affect and as a positive laboratory animal welfare measure.

**3Rs benefits**

Refinement: This work constitutes an attempt to (a) refine the tickling methods and provide further scientific credibility to tickling as a positive social enrichment for laboratory rats by potentially increasing the number of rats that enjoy tickling; (b) encourage wider uptake of tickling by studying the implications of tickling for experimental repeatability.

**Practical benefits**

From our experience there is general interest in tickling as a form of social enrichment but potential users might have reservations. We anticipate that a better understanding of biological consequences of welfare improvement measures could enhance uptake in the wider research community.

**Current applications**

None.

**Potential applications**

We aim to promote a validated playful handling protocol as a refinement to the tickling methodologies currently used.

## Introduction

### Research question: background, importance and relevance

**Figure 1.**
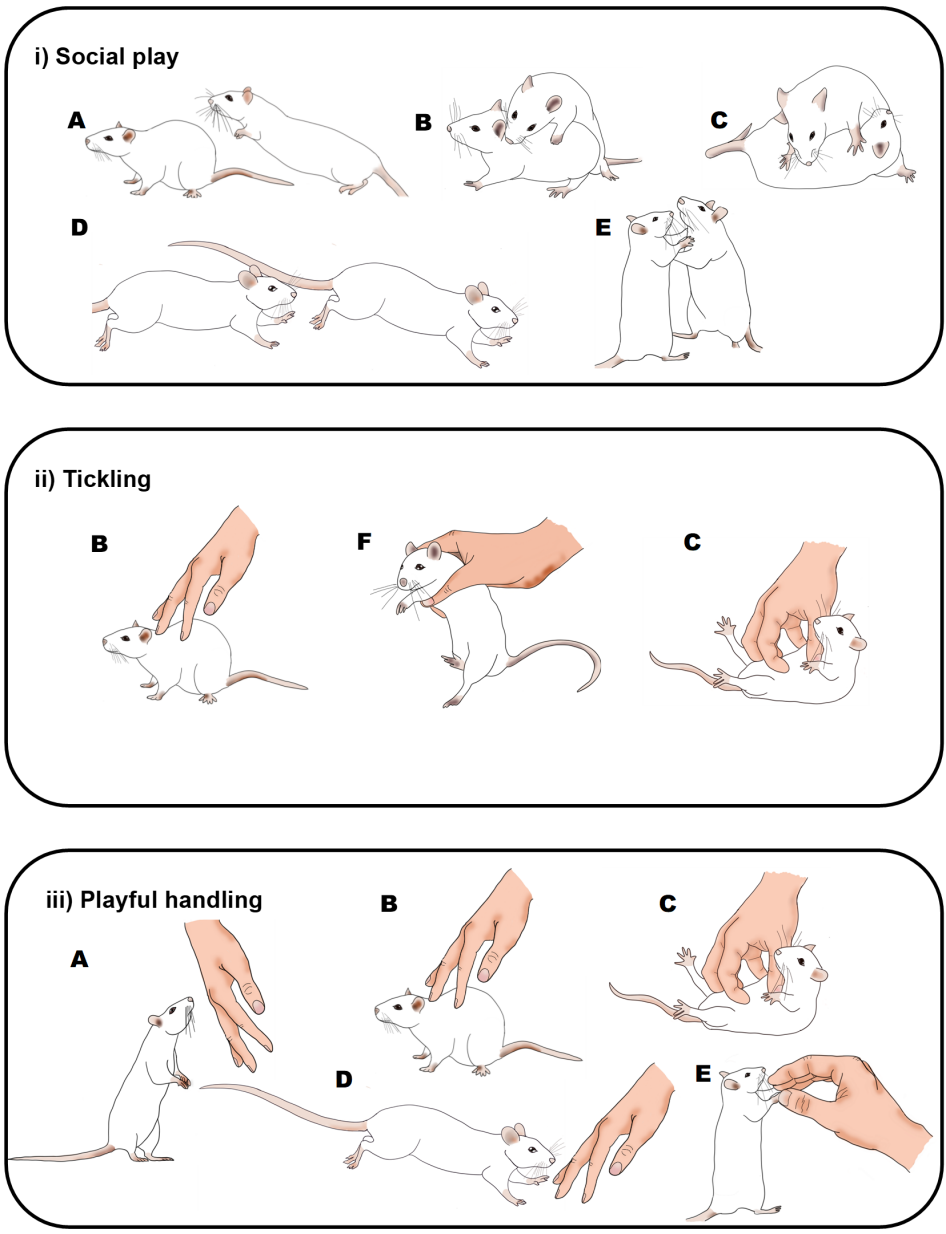
Graphical comparison of playful rat interactions. Illustration of the behaviours seen in rats during: i) social play ( Pellis & Pellis, 2013); ii) the Panksepp variation of tickling ( Cloutier *et al.*, 2018); and iii) playful handling ( Bombail *et al.*, 2019, 2021). Letters indicate rat behaviours that share similar or the same physical characteristics: A) pouncing, B) nape contact, C) pinning, D) chasing, E) boxing and F) flipping. During social play (i) or playful handling (iii), behaviours can occur in any order and do not always occur in each play bout. During tickling, the sequence of behaviour is always B), F) and then C). Drawings by Tayla Hammond.

During 2020 in the UK, 201,600 experimental procedures were carried out on rats, the second most investigated laboratory species ( UK Home Office, 2021). Worldwide there has recently been an increasing emphasis on positive welfare, and the introduction of positive experiences to improve animals’ quality of life ( Lawrence *et al.*, 2019; Makowska & Weary, 2021). The use of manipulations to enhance positive welfare in laboratory animals has been shown not to be a source of variability ( Kentner *et al.*, 2021), and a possible contribution towards improved model validity and data reproducibility ( Loss *et al.*, 2021; van de Weerd *et al.*, 2004; Würbel & Garner, 2007). It is therefore likely that ‘happy animals make good science’ ( Grimm, 2018; Poole, 1997). For instance, positive welfare might reduce stress caused by aversive procedures ( Hinchcliffe *et al.*, 2022). For laboratory rats, a positive handling approach referred to as rat tickling is proposed as an effective and practical approach to positively improve their welfare ( https://www.nc3rs.org.uk/rat-tickling; https://na3rsc.org/rodent-handling/). Rat tickling with the human hand was developed to study positive emotions and ultrasonic vocalisations (USVs) in rats by mimicking playful interactions between rats ( Panksepp, 1998, Figure 1i). The standard tickling protocol involves initial finger contact with the nape of the neck before flipping the rat and gently pinning it in a supine position whilst making rapid finger movements focusing on the ventral surface as used in human tickling ( Cloutier *et al.*, 2018; Figure 1ii).

Research (see Bombail *et al.*, 2021; LaFollette *et al.*, 2017 for reviews) has shown that when rats are tickled by the human hand, they often produce 50 kHz USVs, which are understood to indicate positive affect in rats ( *e.g.*, Barker, 2018, Figure 2i). Other evidence suggests that rats find tickling rewarding as in addition to production of USVs, tickled rats show a reduced latency for approaching the human hand and can be trained to perform operant responses for tickling ( Burgdorf & Panksepp, 2001; LaFollette *et al.*, 2017).

**Figure 2.**
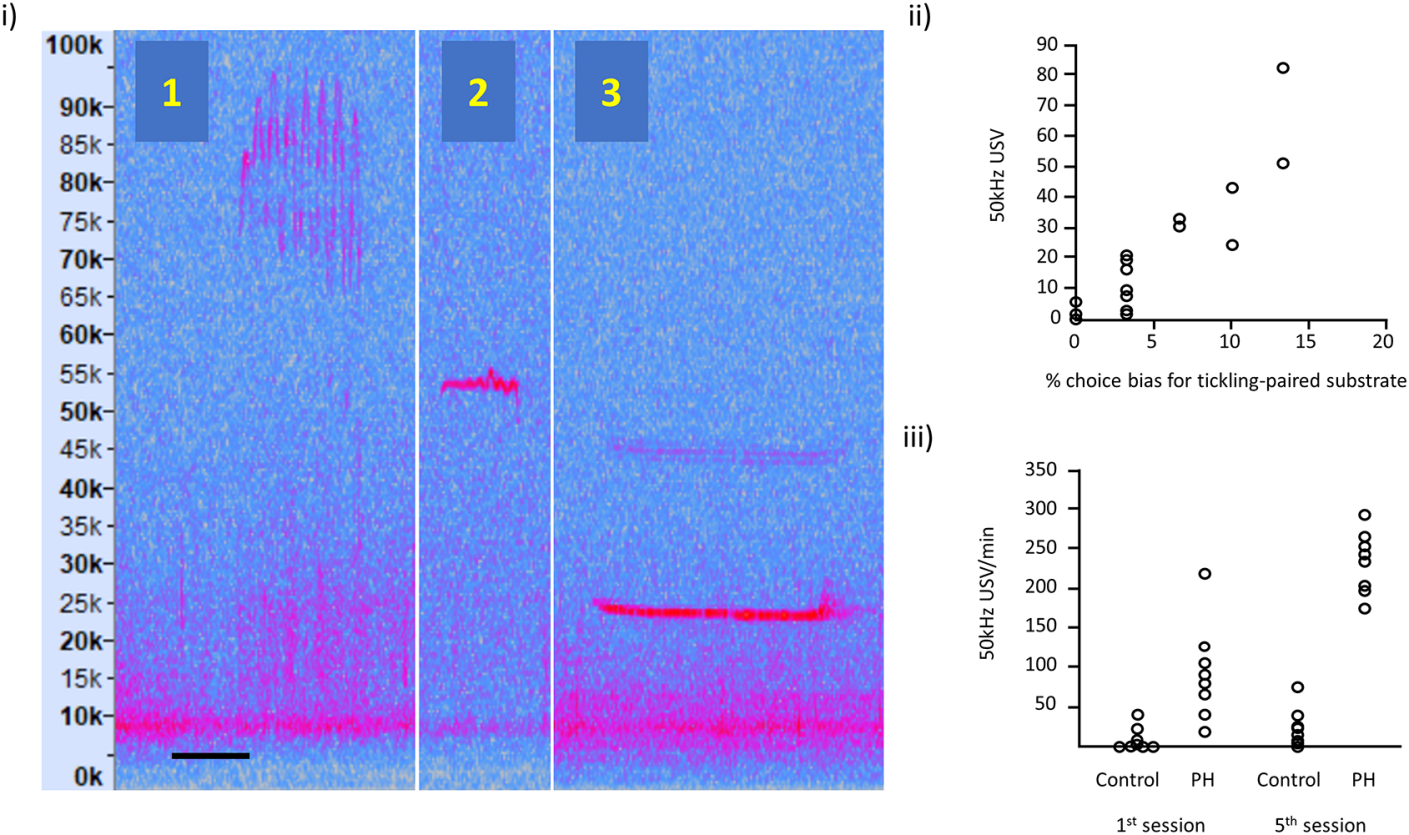
Examples of rat ultrasonic vocalisations (USVs). (i) Illustration of: (1) frequency modulated 50 kHz USVs, (2) flat 50 kHz USV and (3) 22 kHz USV; the spectrograms are taken from our own recordings and these vocalisations are typically expressed in all rats. Vertical axis represents USV frequency (in kHz) and horizontal scale bar represents 50 ms for USV 1 and 2, and 200 ms for USV 3. (ii) Relationship between 50 kHz USV production and an affective bias test (redrawn with data available online, and reproduced with permission from Hinchcliffe *et al.*, 2020). Each data point represents, for each of the 16 rats, the 50 kHz USV response to tickling and the substrate preference in the Affective Bias Test, indicative of the degree of emotional valence. (iii) USVs in playfully handled (PH) rats compared to a control group on the 1 ^st^ and 5 ^th^ session of treatment (redrawn with data available online and reproduced with permission from Bombail *et al.*, 2019). By the 5 ^th^ session, all PH rats showed an increase in USV production in contrast to panel (ii), which used the standard protocol where some rats show little or no increase in USVs.

However, individual responses to the standard tickling protocol indicate that there is a risk that some rats may not perceive tickling to be a positive experience. We summarise that evidence below (see also Bombail *et al.*, 2021).

It is well accepted that rats vary in their response to tickling: The NC3Rs web-site on tickling is clear on this point: “But I hate being tickled; shouldn’t rats hate it too? This is a common concern, and it is true that some rats enjoy it more than others” ( https://www.nc3rs.org.uk/tickling-rats-improved-welfare). We initially developed doubts over aspects of the standard tickling protocol ( Bombail *et al.*, 2019) with its regular use of pinning due to the individual variation in USVs and behaviour we observed in response to this protocol ( *e.g.*, Hinchcliffe *et al.*, 2020; see Figure 2ii). Other work has reported significant levels of individual variation in response to tickling ( LaFollette *et al.*, 2017).

Individual responses to tickling have welfare implications: Rat tickling response is associated with responses indicating affective states ranging from neutral to positive ( *e.g.*, Hinchcliffe *et al.*, 2020), and hence tickling may not possibly always be welfare enhancing. We have shown that USVs in response to tickling are correlated with an independent test of affective state (an affective bias test) indicating that rats vary significantly in their affective response to tickling ( Hinchcliffe *et al.*, 2020). Figure 2ii taken from Hinchcliffe *et al.* (2020) shows variation in USVs in response to tickling correlated with the % choice bias for a tickling-associated digging substrate as the validated measure of affective state. Other evidence supporting a relationship between this naturally occurring variation in response to tickling measured as USV production and welfare outcomes, such as affective states, can be found in work from Rygula *et al.* (2012) and Mällo *et al.* (2007, 2009).

We therefore propose that individual variation in response to the standard tickling protocol is a significant issue when promoting the widespread use of tickling as an approach to improving rat welfare. Due to our experiences using the standard tickling protocol we have developed a playful handling (PH) protocol intended to better mimic the dynamic nature of play, giving the rat more choice over how it is tickled ( Bombail *et al.*, 2019, Figure 1iii). The PH protocol is described by Bombail *et al.* (2019) as the handler using one hand to touch, tickle, and play with the rat for 20 second periods interspersed with 20 second pauses. During the active periods, the handler mimicked the rough-and-tumble play seen in adolescent rats, with the hand tickling, chasing and pinning the rat, depending on its response. Our data suggest that the PH protocol resulted in a more uniform increase in USVs across individuals relative to the standard protocol. Figure 2iii adapted from Bombail and colleagues ( Bombail *et al.*, 2019) shows USVs in playfully handled rats *vs.* a control group (who did not play in the day of recording) on the 1st and 5th session of treatment. By the 5th session, all playfully handled rats showed an increase in USV production in contrast to Figure 2ii, which used the standard protocol and where some rats show little or no increase in USVs.

We conclude that this evidence calls for a scientifically based refinement of tickling to ensure that the positive effects of tickling are maximised and that individual differences in response to tickling are minimised with benefits to rat welfare and the reproducibility of research where tickling is applied as a treatment or more generally as an enrichment. In this report, we define tickling as the standard protocol ( *e.g.*, Cloutier *et al.*, 2018) and, although it was initially coined as an equivalent term to tickling ( Cloutier *et al.*, 2018), PH as the approach outlined in Bombail *et al.* (2019).

### Hypotheses

PH consists in mirroring playful behaviour by allowing for more flexibility between the human handler and the rat and consequently we hypothesise PH will induce a more homogeneous positive affective response. We also hypothesise PH will lead to reduced variability of biological outcomes.

*Confirmatory Hypotheses:* ( *Confirmatory Hypothesis* 1) We hypothesise that rats exposed to handling protocols that maximise playful interactions ( *i.e.*, where pinning number per session decreases) will show an overall increase in total 50 kHz USVs as an indicator of positive affect. ( *Confirmatory Hypothesis* 2) We also predict that 50kHz USV measures will be less variable in treatments with less pinning.

*Exploratory Hypothesis 1:* We hypothesise that the behavioural and physiological changes associated with alterations in PH experience will be less variable when rats are exposed to handling protocols that maximise playful interactions (where pinning number per session decreases). There is no previous data on this question, we cannot generate power calculations and pre-register this hypothesis, this is therefore exploratory work.

### Study timeline

**Figure 3.**
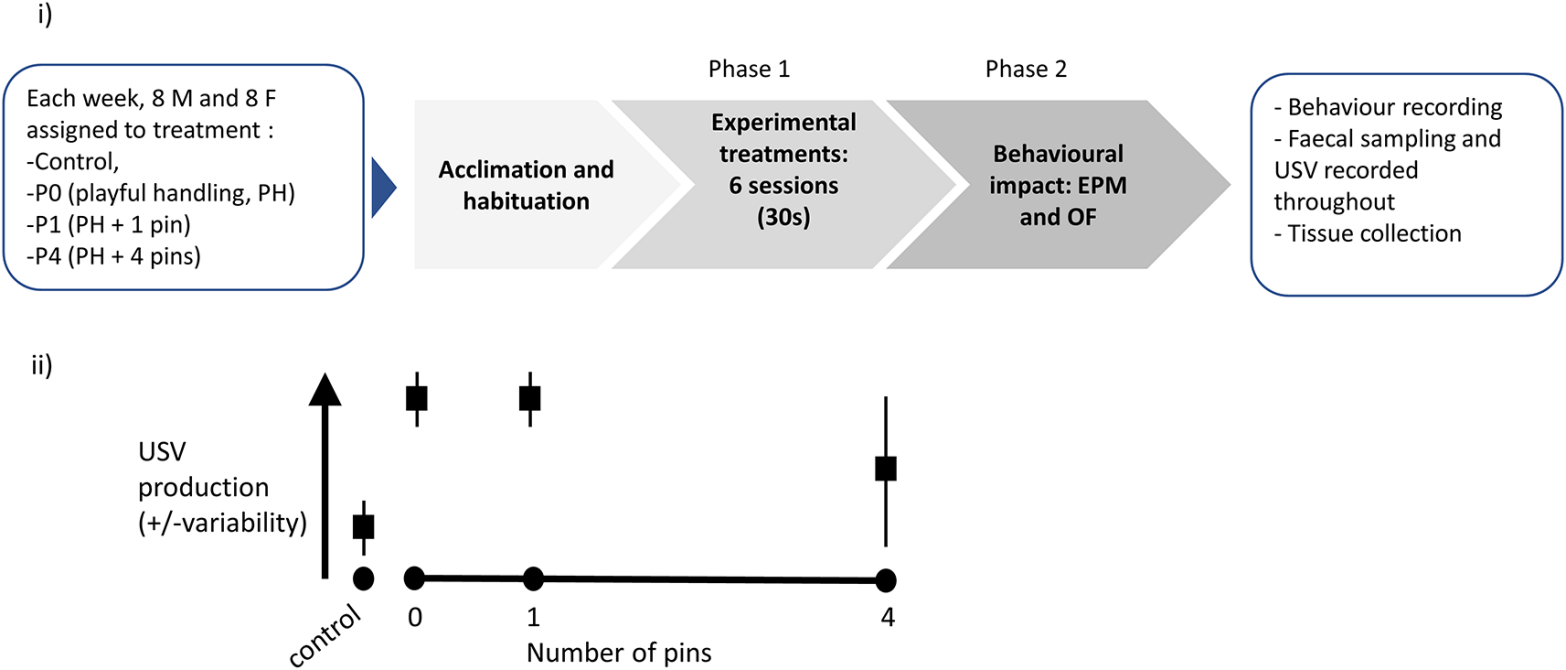
Experimental plan and graphical illustration of hypothesis. (i) Graphical representation of the experimental protocol. Upon reception, each cohort (n=4 cages, with two males per cage and n=4 cages, with two females per cage) is attributed to cage and treatment, then left to acclimatise for a week. Phase 1 consists of exposure to six sessions (30 seconds daily) of either Control, P0, P1 or P4 treatment (varying in the number of pins administered relative to background playful handling; see Objective 1 above). Finally, the rats are behaviourally tested in Phase 2, using Elevated Plus Maze (EPM) then Open Field (OF), and sacrificed for tissue collection. A step-by-step protocol is outlined in box 1. (ii) Prediction against which our confirmatory hypothesis will be tested: we predict P0 and P1 treatments will lead to higher and less variable induction of ultrasonic vocalisation (USV) production, compared to P4.

The diagram presented in Figure 3 synthesises the timeline for each three-week long cohort studied. The experimental part of this study will be conducted using eight such cohorts, each made up of 16 animals. The timing of these cohorts will be staggered, so experimental cycles overlap, with 16 animals brought into the facility every week, following successful peer review of this Stage 1 Registered Report submission. A step-by-step protocol is available in Box 1. The experimental work for this study should be completed by 21 December 2022, the data analysis should be completed by 1 July 2023.

Box 1. **Step-by-step protocol.**

Prior to the experiment and from day 1 to day 6 of the first cohort, various optimisation and preparation test recordings will be carried out, this will result in a general level of noise as throughout the experiment (when several cohorts are housed and tested simultaneously)

Day 1. Reception of rats and group formation (morning).

- Temporary mark on tail,
- Weigh each rat,
- Form eight cage pairs based on : (1) sex, (2) provenance (always housed with an individual from a different litter), (3) similar cage weight (monitor total treatment group average as the values must balance by the 8 ^th^ cohort).

Day 1 to day 6. Rats habituate to facility environment.

Day 7. Habituation to PH testing arena.

- Collect faecal pellets for faecal corticosterone metabolite (FCM) analysis. Weigh rats. Habituation 1: in the morning (start of dark phase), for each of the eight cages, transfer pair into the play arena and leave together for five minutes. Remove any faeces from arena if necessary.
- Habituation 2: in the afternoon, for each of the eight cages, transfer pair into the play arena and leave together for five minutes. Remove any faeces from arena if necessary.

Day 8. Habituation to PH testing arena.

- Preparation of the next cohort (Day 1)
- Habituation 3: in the morning, for each of the 16 individuals, place into the play arena and leave for five minutes. Remove any faeces from arena if necessary.
- Habituation 4: in the afternoon, for each of the 16 individuals, place into the play arena and leave for five minutes. Remove any faeces from arena if necessary.
- Cage bedding is changed.

Day 9 to day 11. Experimental treatment.

- The cage will be moved close to the testing arena five minutes prior to the first of the pair of rats being introduced in the test arena. Both rats will be filmed during this anticipatory phase, to investigate their behaviour.
- From each cage, a rat taken individually into the arena (order of passage pseudo-randomised), recorded for a one min anticipation phase and treated according to the schedule (control, P0, P1 or P4) for 30 seconds. Ultrasonic vocalisation (USV) are recorded. Arena wood shavings stirred between each animals (for odour homogenisation).
- Following this treatment, rats will be gently picked up and returned to the home cage. Their cage mate will then be immediately placed in the arena for testing and the same tickling treatment repeated.
- The home cage is covered with a cardboard screen, behind the plastic curtains protecting other cages from emotional contagion *via* USV.
- Two hours later, each cages are placed back on the housing rack.

Days 12 and 13 are the weekend, animals are undisturbed except for welfare checks.

Day 14 to day 16. Experimental treatment.

- Morning of Day 14 only: Weigh rats
- Day 15 only: preparation of the next cohort (Day 1)
- The cage will be moved close to the testing arena five minutes prior to the first of the pair of rats being introduced in the test arena. Both rats will be filmed during this anticipatory phase, to investigate their behaviour.
- From each cage, a rat taken individually into the arena (order of passage pseudo-randomised), recorded for a one-minute anticipation phase and treated according to the schedule (control, P0, P1 or P4) for 30 seconds. USV are recorded. Arena wood shavings stirred between each animals (for odour homogenisation).
- Following this treatment, rats will be gently picked up and returned to the home cage. Their cage mate will then be immediately placed in the arena for testing and the same tickling treatment repeated.
- The home cage is covered with a cardboard screen, behind the plastic curtains protecting other cages from emotional contagion *via* USV.
- Two hours later, each cages are placed back on the housing rack.

Day 17. Collect faecal pellets for FCM analysis. Elevated Plus Maze (EPM) testing late morning (middle of dark phase). Standard EPM protocol, EPM will be cleaned between each animal.

Day 18. Open Field (OF) testing.

- Rats are tested in the OF in the morning, Standard OF protocol, OF will be cleaned between each animal.
- Rat are weighed
- Rats are injected with an overdose of pentobarbital and tissues sampled for molecular studies to investigate physiological impact of experimental treatments. Collect faecal pellets for FCM analysis.

## Methods

### Basic methodological framework/identification strategy

Our experimental design is a pseudorandomised control trial where every week, each of the eight cages of two male or two female rats will be assigned to one of four treatments (control, P0, P1, P4). Treatment allocation is described below. Within a cage, individuals cannot be considered independent, as they might influence each other in response to the treatment through emotional contagion, we will therefore fit the cage as a random effect for all treatments and both sexes.

### Ethical approval

All animal work will be carried out at the Roslin Institute, Edinburgh, U.K., in accordance with the U.K. Animals (Scientific Procedures) Act 1986 and reported in compliance with the ARRIVE guidelines ( Bombail, 2022). This work does not involve aversive procedures (and is deemed less harmful and distressful than skilled insertion of a hypodermic needle according to good veterinary practice) and therefore not legally required to have UK Home Office approval. As recommended ( Olsson *et al.*, 2022), we collectively discussed welfare and ethical considerations with the Named Animal Care Welfare Officers, veterinarians and scientists involved in the study. Following consideration by the University of Edinburgh’s Animal Welfare and Ethical Review Body (reference NC/W001209/1, 26 July 2022, Establishment number X212DDDBD), it was confirmed the work was subthreshold to regulation under the U.K. Animals (Scientific Procedures) Act 1986, and the committee approved it as detailed in this paper. This study was also approved by the Veterinary Ethical Review Committee of the Royal (Dick) School of Veterinary Studies, University of Edinburgh (reference 92.22, 2 September 2022).

### Experimental animals

Practical and financial constraints require that we limit this study to a single strain. We will use Wistar Han rats (Research Resource Identification, RRID:RGD_2308816) purchased from Charles River Laboratories (CRL, Margate, UK). This outbred strain has been shown to be reliably responsive to tickling and PH ( Bombail *et al.*, 2019; Hammond *et al.*, 2019) and is one of the commonly used strains for such experiments (23% of rat tickling studies used Wistar, LaFollette *et al.*, 2017). We will purchase the rats from CRL (16 at a time), aged 3-4 weeks and will complete the research prior to their puberty around 7 weeks of age. LaFollette *et al.* (2017) found that over 60% of papers on tickling used rats within this time window. We have also recently found evidence of sex differences with female rats producing more USVs than males in response to tickling ( Tivey, 2022) and each cohort will consist of equal numbers of males and females.

We aim to carry out the experiment in eight cohorts of n=8 cages (with two rats per cage) and equal numbers of males and females in each cohort. A total of 128 rats (64 males and 64 females) will be used in this study, in accordance with our power analysis detailed below.

To the greatest extent possible we will aim for a matching number of males and females coming from the same litter. Depending on availability, the number of litters sampled will be two to four per cohort (we are aiming for two litters per cohort, four males and four females from the same litter). Rat treatment allocation will be spread evenly across all litters to prevent bias due to genetic background and early life experience ( *i.e.*, each of the four individuals from each sex will be assigned to each of the four treatments). We have arranged for CRL to only provide animals from experienced parents (sire/dam will have been sired/nursed at least one previous litter). This will reduce variability in pup early life environment caused by differences in parental experience.

### Acclimation, treatment allocation, housing and husbandry

On arrival at the Roslin Institute facility, the rats will be housed in a bespoke behaviour research room with an average room temperature of 22°C and relative humidity of 43%. The rats will be on a 12 h:12 h reverse light cycle (lights off at 06:00), to allow testing of the rats during their dark photoperiod when they are most active and to accommodate the preference of albino rats for lower light intensities (see one of our previous reports ( Hammond *et al.*, 2019)). All treatments and behavioural tests will be carried out between 8:00 and 16:00 (2 hours from lights on or off). The allocation of rats to cages will be carried out in the following order: sex, litter of origin and body weight. Rats will be weighed and allocated to home cage as pairs of the same sex (but never from the same litter). As much as feasibly possible, we will aim to house together rats of similar weight. Pairs will be assigned to a treatment using a random list generator. We aim to homogenise weights between groups. For each sex, rat weight will be averaged for each treatment, and to avoid potential bias caused by uneven distribution of body weight between treatment groups, adjustments in treatment distribution will be made to balance evenly weights across treatments ( *e.g.*, we will assign heavier pairs of animals to treatments that previously contained lighter animals). Rats will be left to acclimatise for up to six days prior to handling.

The home cages are made of clear plastic with a metal mesh open-top lid (l × d × h: 612 mm × 435 mm × 216 mm), filled with 2 cm-deep woodchip bedding, shredded paper, a 20 cm-long red plastic tunnel, a 10 cm wooden chewstick and a 2 cm diameter wooden ball as enrichment (all as per normal practice in our animal facility). This size cage allows animals to rear and stand on their hind limbs. Rats will have *ad libitum* access to standard rat chow (current supplier: Teklad Global Rodent Maintenance Diet (14% protein); Envigo, UK) and tap water. The cages will be distributed across four tiers of a standard cage rack. Due to the varying lux levels and other potentially confounding variables, position of the home cages in the rack will be randomised across treatments (as in Hammond *et al.*, 2019). The rats will be checked daily, and nitrile gloves worn when handling the animals. To minimise handling stress, rats will be picked up gently by holding them behind their forelegs and then cupping them with both hands. Cage bedding will be changed once, halfway through the experiment (on the afternoon of day 8), according to the routine protocol (a small volume of soiled litter without faeces will be kept in the cage to maintain a familiar olfactory environment). Body weight will be measured routinely across the study (on days 1, 7, 14, 18).

### Experimental treatments

A unique male experimenter (with prior experience in all rat playful interactions tested) will perform all treatments and behavioural tests. All experiments will be performed in the same room as where the animals are housed, as in previous studies ( *e.g.*, Hammond *et al.*, 2019). Using curtains and physical distance, we will prevent emotional contagion phenomena involving USV ( *i.e.*, we have confirmed USV cannot be detected by other animals, using our recording equipment). Rats are used to a degree of audible activity in the room throughout the experiment. The play arena, placed behind fabric curtains 6 m away from the home cages, will be a Plexiglas square (60 cm) of depth 25 cm, filled with 3 cm-deep woodchip litter in which Wistar rats have played previously (over 200 sessions by 16 males rats took place by the start of the experiment). We keep the same litter for all experiments and remove the rare faeces produced by the animals (in our experience, exclusively produced during the habituation sessions). Litter is regularly stirred between animals. We use this method to guarantee the litter contains odorants from several rats, thereby standardising, diluting and minimising the impact of individual olfactory cues. This can be replicated in other laboratories by using a mixture off soiled litter and clean litter. Following experimental treatment (described below), cages will be left, loosely covered with carboard screens, for 2 h in an area behind plastic curtains (a switched off Lateral Air Flow cabinet), before being returned to their usual home cage rack position. In the absence of this ‘cool down’ period, in our experience a marked rise in USV production over time can be otherwise be measured in control rats ( Lam, 2017).

The rats will be habituated to handling and the experimental recording arena, to lessen any anxiety or fear due to novelty, as negative affective states inhibit play behaviour (reviewed in Ahloy-Dallaire *et al.*, 2018). For habituation to the treatment arena, rats will be moved in their cage to be beside the treatment arena before being lifted gently into the arena, and left there to explore, first as a pair of cage mates (2 × 5 minutes), on the first day, and then alone (2 × 5 minutes) on the second day.

The aim of PH is to give all rats a similar amount of hand contact (we approximate 70–80% of human-animal interaction time, *e.g.*, about 24 out of 30 seconds), while being responsive to differences between individuals. The similar amount of physical contact for all individuals is important, since activation of the cortical area in response to touch has been shown to influence USV production ( Ishiyama & Brecht, 2016). When not touching the rat, the hand can also approach the animal while the hand is in contact with the wood shavings, to provide sensory cues consistent with a play partner approaching ( *e.g.*, about 6 out of 30 seconds).

Another key component of PH is to be responsive to individual differences, which can range from more playful individuals who will quickly move (accelerate or turn) to face the hand, and display playful behaviour ( *e.g.*, joy jumps ( Reinhold *et al.*, 2019)) to less playful individuals who move less and more slowly and show little or no play behaviour. An example of PH with a responsive individual is available to watch online here. PH starts with presentation of the back of the experimenter’s hand for a brief sniff and exploration by the rat. The experimenter then starts playing with the rat using a rapid tickling finger motion on the sides, back and front of the rat (alternating region). The experimenter should focus on being playful (unexpected moves, fast change of hand movements, change of pace) rather than simply the physical act of tickling ( *e.g.*, Figure 1iii).

Pinning consists in approaching the rat during PH as described above. Thumb and middle fingers are then placed behind the front legs, and the animal is gently but swiftly moved onto its back, and upon landing, while the rat is pinned down, it is tickled on the front of its thorax and abdomen involving rapid tickling movements of fingers ( *e.g.*, see Cloutier *et al.*, 2018).

In both PH and standard tickling, movements should reflect that rat play is boisterous ( Cloutier *et al.*, 2018) with finger pressure being firm and movements fast.

### Objective 1: Testing protocols varying in PH and pinning events against USV as a measure of affective state

*Confirmatory Hypotheses:* (Confirmation Hypothesis 1) We hypothesise that rats exposed to handling protocols that maximise playful interactions ( *i.e.*, where pinning number per session decreases) will show an overall increase in total 50 kHz USVs as an indicator of positive affect. (Confirmation Hypothesis 2) We also predict that 50 kHz USV measures will be less variable in treatments with less pinning.

Objective 1 will be addressed in Phase 1 of the experiment (see Figure 3).

*Experimental design:* The aim of this objective is to refine the standard tickling protocol based on the rats’ affective response to treatments that vary in PH and number of pins. All rats will be exposed to six sessions of experimental human-animal interaction treatment, over eight days, interrupted by a two-day weekend break. We have chosen a standard session length of 30 seconds that reflects our previous work ( Hinchcliffe *et al.*, 2020) and also the practical reality that if tickling is to be used widely in practice it needs to be applied in a time efficient manner ( LaFollette *et al.*, 2019). The order of testing of cages, and rats within cage will be pseudo-randomised (a sequence for cage and individuals will be generated from a random list generator). The cage will be moved close to the testing arena five minutes prior to the first of the pair of rats being introduced in the test arena. Both rats will be filmed during this anticipatory phase, to investigate their behaviour.

Rats will be removed individually from the home cage, placed into the handling arena and, following an additional one-minute recording of anticipatory play behaviour, the experimental treatments applied:

P0: ‘PH’, where zero pins will be applied; the experimenter using one hand will touch, tickle and chase the rat in a manner that mimics rough and tumble play ( Bombail *et al.*, 2019; Figure 1iii).

P1: One pin will be applied at approximately 15 seconds from the start of the tickling session. The rat will be held and flipped into a supine position and the experimenter will gently pin the rat in this position whilst moving their fingers quickly and vigorously but gently on the belly, as is commonly used in human tickling. The pin will last for between 2–4 seconds and the rat will be allowed to right itself at the end of pinning ( Cloutier *et al.*, 2018; Figure 1ii). On either side of the pin the experimenter will engage in PH as in P0.

P4: Four pins will be applied from five seconds into the tickling session (four pins being the number delivered during a 15 second pinning session in the standard protocol ( Cloutier *et al.*, 2018)); pins will be applied as in P1 and the rat will be allowed to right itself after each pin with a short gap before the next pin. On either side of the pinnings the experimenter will engage in PH as in P0.

Control: We will apply the same control treatment as in our previous work ( Bombail *et al.*, 2019; Hammond *et al.*, 2019) where experimenter places their hand against the inside of the arena for the entire session.

Following this treatment, rats will be gently picked up and returned to the home cage. Their cage mate will then be immediately placed in the arena for testing and the same tickling treatment repeated.

### Data collection

We will record the behaviour during the tickling session using a digital HD camcorder. We will record USV using a high-quality microphone designed for recording ultrasonic vocalisations produced by bats (Pettersson M500-384 USB Ultrasound microphone) and Audacity recording software. The microphone will be placed over the centre of the arena, pointing downwards at the arena floor. Total USV per unit of time will be quantified by spectrogram analysis.

Measures: (i) Vocalisations will be recorded during the handling sessions as in our previous work ( Bombail *et al.*, 2019; Hammond *et al.*, 2019) and categorised into 50 kHz USVs and 22 kHz USVs; (ii) We will also record other quantitative behavioural responses to tickling as per our previous work ( Hammond *et al.*, 2019).

Our primary response variables for Confirmatory Hypotheses 1 and 2 will be:

(a) Total 50 kHz USV: We will analyse 50 kHz USV as indicators of positive affective state ( *e.g.*, Barker, 2018; Brudzynski, 2013). We define 50 kHz USV as calls with a peak frequency between 30 and 80 kHz and a short duration between 10–400 ms (based on Brudzynski, (2013) and our own empirical observations). We predict 50 kHz will be significantly increased in P0 and P1 treatments, where pinning is minimised, relative to P4, where play is restricted relative to pinning, and the Control treatment, where there is no play.
(b) Variability of 50 kHz USV production: we will compare individual variability across the different treatments. We predict a greater individual variability in positive affective states in treatments that maximise pinning.

Our secondary response variables will be:

(a) Categorisation of 50 kHz calls: we will analyse sub-types of USV following segregation based on their acoustic properties, according to published criteria ( Wright *et al.*, 2010). We will investigate whether certain USV types might be preferably associated with certain treatments.
(b) Anticipatory solitary play behaviours: We will also analyse anticipatory play behaviour, as this has been shown to be an indicator of positive affect. We will use the ethogram in Table 1, based on observations from our earlier work ( Bombail *et al.*, 2019; Champeil-Potokar *et al.*, 2021; Hammond *et al.*, 2019).
(c) Behavioural transitions: we will analyse the number of behavioural transitions (including play) that occur prior to treatment, as indicators of reward anticipation ( Anderson *et al.*, 2015).
(d) 22 kHz USV: We will also analyse 22 kHz USVs as indicators of negative affective state ( Brudzynski, 2013). We define 22 kHz USV as calls with a frequency between 20–30 kHz, and a long duration between 500–3000 ms ( Brudzynski, 2013). We predict overall very low number of events, clustered in treatments that maximise pinning.

**Table 1.** Ethogram for study of anticipatory behaviour (based on our previous studies and taken from Bombail *et al.* (2019), Champeil-Potokar *et al.* (2021) and Hammond *et al.* (2019).

Behaviour	Description
Move	Locomotor behaviour, *e.g.*, mid rearing or going down, or travelling horizontally
Up explore	Sniffing and exploring, while rearing
Down explore	Sniffing and exploring, interaction with litter, eating (litter or coprophagia)
Dig litter	Dig litter
Immobile	Lack of perceivable movement
Grooming	Includes partial grooming sequences
Solitary play	Seemingly spontaneous movement involving at least two hops, where a hop involves all four paws leaving the ground at the same time; can occur from stationary or during locomotor movement; behaviour performed not in the direction of the cage mate (play partner) during a play bout or as an evasion response to being chased by a play partner
Social play	Described in Figure 1c. One rat pounces or rubs on the partner to solicit play, resulting in the partner either chasing the soliciting rat, rearing (the pair makes rapid pawing movements at each other) or rotating to where one rat is pinned onto its back with the other standing over it.

*Analyses*

Data will be analysed in R through General Linear Mixed Models using the lme4 package ( Bates *et al.*, 2015), to test our Confirmatory Hypothesis that indicators of positive affect are increased in treatments P0, P1 where PH is increased and amount of pinning reduced, relative to P4 and Control ( *i.e.*, no play) treatments. Our fixed effects are tickling treatment (Control, P0, P1, P4), sex (male, female) and the tickling*Sex interaction. In addition to testing differences in average USV we will also test whether treatments P0, P1 have reduced the variability of response relative to P4 as we anticipate (using the Levene’s test for equality of variance or equivalent). As individuals from a pair cannot be considered independent, as they might influence each other in response to the treatment, we will fit the cage as a random effect for all treatments and both sexes.

### Objective 2: Testing the behavioural and physiological effects of protocols varying in PH and pinning events

*Exploratory Hypothesis:* We hypothesise that the behavioural and physiological consequences of tickling will be less variable and more repeatable when rats are exposed to handling protocols that maximise playful interactions and where pinning episode number per session decreases. Due to the paucity of data on the impact PH might have on the variables we will measure, we cannot generate power calculations and pre-register this hypothesis, this is therefore exploratory work.

Objective 2 will be addressed in Phase 2 of the experiment (see Figure 3).

*Experimental design:* In Phase 2, the next day after the final playful or control interaction, rats will undergo behaviour testing with Elevated Plus Maze (EPM) and Open Field (OF), their order of passage will be pseudo-randomised. The EPM and OF test consist in quantifying exploration behaviour in 2 different structures, that both consist in an area with open spaces (the centre of the OF and the open arms of the EPM) and an area with more enclosed spaces (the periphery of the OF and the closed arms of the EPM). For rats, this ambiguity creates a conflict between the thigmotaxis (preference for proximity to walls) and the desire to explore open spaces, which is used to assess the emotional response to those novel experiences, and to test for anxiety levels. Following these tests, the rats will be humanely killed by anaesthetic overdose and tissues collected for analysis. Post mortem tissue measures will be critical to assess the impact of our treatments on variability and repeatability of commonly used measures of physiological function. Faecal pellets will be collected on days 17 (morning) and 18 (post cull) for non-invasive corticosterone metabolite analysis, in comparison to pellets collected before treatment starts on day seven.

*Measures:* (i) Behavioural measures of response to OF and EPM ( Ethovision) will be collected to quantify anxiety behaviour prior to culling; (ii) Physiological measures of stress and inflammation: Faecal samples will be collected on day 18, at the end of Phase 2 (and compared to levels at the start and end of Phase 1) and assayed for corticosterone ( Lepschy *et al.*, 2007) using a commercially available corticosterone ELISA (Enzo LifeSciences). Point of cull plasma will be assayed for a set of inflammatory markers including the pro-inflammatory cytokines IL1a, IL1b, IL2, IL6, IFNγ and TNFα and the anti-inflammatory cytokines IL4, IL10, IL13, using a magnetic bead based multiplex ELISA (FIRELEX); our selection of inflammatory markers includes cytokines known to be sensitive in humans to negative and positive affect ( Anisman & Merali, 2003; Stellar *et al.*, 2015); (iii) Other tissues such as the brain, pituitary gland, heart, liver, spleen and gut will be collected at culling and flash frozen on dry ice and stored at -80 ^o^C for future analyses.

Our primary response variables will be:

a) EPM: increased markers of open arm exploration (% time and distance exploring the open arm) reflect reduced anxiety, based on rat aversion of open spaces ( Kraeuter *et al.*, 2019).
b) OF: decreased markers of thigmotaxis (% time spent and distance covered close to the peripheral walled area) reflect decreased anxiety, based on rat aversion of open spaces. Markers of activity (rearings, total distance covered, bouts of locomotion) will also be recorded since, although interpretation can be challenging ( Denenberg, 1969), they do reflect arousal upon OF arena exploration.

Our secondary response variables will be:

a) Physiological and inflammation related variables: we will analyse markers regulated in affective states, and their variability around the treatment mean/median. We expect P0 and P1 to be more effective at inducing anti-inflammatory cytokines and reducing pro-inflammatory cytokines, and faecal corticosterone ( Anisman & Merali, 2003; Lepschy *et al.*, 2007; Stellar *et al.*, 2015), in comparison to P4 and control treatments.
b) Body weight: we will keep a record of weight as an indicator of general body condition, we do not expect any treatment-related changes, as per our previous studies ( Bombail *et al.*, 2019; Hammond *et al.*, 2019).

*Analyses*

Using the same approach as described for Objective 1, data will be analysed in R using Generalised Linear Mixed Models to ascertain the effects of tickling treatments and sex on behavioural and physiological measures. To test our Exploratory Hypothesis that the biological consequences of tickling will be less variable among rats and more repeatable within rats when rats are tickled using more playful protocols (P1, P0), we will analyse the residual variance from the undertaken models and explore intraclass correlation coefficients of combined random and fixed effects.

### Diagram of the experimental plan

A diagram of the experimental procedure is presented in Figure 3i. We also present a graphical representation of our predicted results, which will be tested in our experiments ( Figure 3ii).

### Further exploratory analyses

Brain tissue will be collected for future analysis of neurobiological consequences of the handling treatments. When funding and collaborations can be raised, it is our intention to further explore the biology of positive affective states through analysis of physiological functions that are associated with emotional experiences ( *e.g.*, immunology or metabolism). We will also analyse USV production in relation to specific USV types ( Wright *et al.*, 2010) using spectrograms from our recordings, with a view to correlating specific USV types to treatments and behaviours.

### Statistical analysis

Specific aspects of data analysis are described in the descriptions of objectives 1 and 2 above. Prior to analysis, we will visualise and test the data for the assumptions of the cognate statistical approaches (data distribution normality or heteroskedasticity), and when necessary, we will use non-parametric alternatives or data transformation. To account for potential type I errors that may arise from multiple comparisons, we will use Bonferroni correction or equivalent (depending on data distribution). We will consider the p-value threshold of 0.05 as significant in our statistical comparisons.

*Power analysis:* Based on our previous data, the most variable response measure is total 50 kHz USVs with data taken from our own and other work ( LaFollette *et al.*, 2018; Tivey, 2022) where treatments come closest to matching our proposed treatments. We have used these data to provide us with means per primary fixed effect combinations ( *i.e.*, treatment x sex) and pooled standard deviations, with resulting simulations based on the smallest effect size (0.09). Power analysis and sample size estimations were conducted using GLIMMPSE software. The desired power was set at 0.8, the type I error rate at 0.05, the means scale factor was set at 1.0 and the variability scale factor at 1.3 using the Hotelling Lawley Trace test ( Hotelling, 1931). Simulations incorporated the non-independence of rats within a cage (cage fitted as a random effect), *via* a calculated intraclass correlation of 0.17 based on previous data ( LaFollette *et al.*, 2018; Tivey, 2022). Additionally, repeated measures over six tickling treatment events/exposures were included in the power analysis. Our fixed effects are treatment (Control, P0, P1, P4), sex (male, female) and the tickling*Sex interaction. Based on simulations detailed above, a sample size of n=16 rats ( *i.e.*, n=8 cages) per treatment and sex combinations, or n=64 total experimental units (128 total animals), provides a power of 0.829. In the spirit of transparency, we will report effect sizes and uncertainty in our analyses.

*Blinding:* We will not be able to blind handlers during the preparatory and experimental phases, as the handlers need to know which handling method they will be using with each rat. We will also not be able to blind collection of quantitative behaviour during tickling sessions, as it will be obvious which handling method is being used in each session. We will blind all other aspects including collection of USVs, analysis of physiological samples, and collation and analysis of behavioural data.

*Outlier extraction:* We are aware of the high variability in USV response among rats, and are experienced in recording it, and therefore will not exclude rats based on their USV production. For physiological markers we will use data points within the dynamic range of the assay and will be checking replicate quality. In the unlikely event of outlier removal (based on the Grubb’s test ( Grubbs, 1969)), this will be explicitly described in the result section. Additionally, should any animal be excluded from the study for health reasons (e.g. injury or infection), we will report it in order to achieve full transparency about the fate of our experimental rats.

## Study status

We are currently awaiting publication of this peer-reviewed protocol to start experimental data collection.

## Study limitations

We will assign animals to treatments according to preset criteria to homogenise factors we assume might affect treatment response (sex, litter of origin and social cage environment, body weight). Our pseudo-randomisation procedure will therefore be more deterministic than the computer-run randomisation algorithm; cryptic biases might emerge upon data interpretation. Additionally, the experimenter will be aware of the treatments administered as the rats are handled. He will therefore not be blinded to the treatments. This is limited by the lack of discernible patterns (order of passage pseudo-randomisation) and impossibility to memorise treatments for each 64 cages at the time of analysis. Finally, we accept use of a single male experiment constitutes a limitation, especially in the light of recent reports about experimenter sex affecting rodents. We have to start somewhere, and although this will not the ultimate testing of the hypothesis, this will be a first attempt that might next be transposed to other laboratories and rat strains.

## Data availability

### Underlying data

No data are associated with this article.

### Reporting guidelines

Figshare: ARRIVE guidelines for ‘Stage 1 Registered Report: Refinement of tickling protocols to improve positive animal welfare in laboratory rats’. https://doi.org/10.6084/m9.figshare.20914993 ( Bombail, 2022).

Data are available under the terms of the Creative Commons Zero “No rights reserved” data waiver (CC0 1.0 Public domain dedication).
