# Supplementary material for: Stage 1 Registered Report: Refinement of tickling protocols to improve positive animal welfare in laboratory rats
Source: F1000Res. 2022 Nov 18;11:1053. Originally published 2022 Sep 15. [Version 2] doi: 10.12688/f1000research.125649.2 (PMC9811030; doi:10.12688/f1000research.125649.2)
Supplement: Peer review history

# Peer review: Reviewer response for version 2

**DOI:** 10.5256/f1000research.140907.r156626

**Published:** 2023-01-04

**Authors:** Theresa M. Kisko

**Affiliations:** 1 Social and Affective Neuroscience – Brain and Cognition Research Unit, KU Leuven, Leuven, Belgium

I have no further comments.

Is the study design appropriate for the research question?

Yes

Are the 3Rs implications of the work described accurately?

Yes

Have the authors pre-specified sufficient outcome-neutral tests for ensuring that the results obtained can test the stated hypotheses, including positive controls and quality checks?

Yes

Is the rationale for, and objectives of, the study clearly described?

Yes

Are sufficient details of the methods provided to allow replication by others?

Yes

Are the datasets clearly presented in a useable and accessible format?

Yes

Are a suitable application and appropriate end-users identified?

Yes

Reviewer Expertise:

Social play behaviour in rats, Ultrasonic vocalizations, Social and affective behaviours and communication.

I confirm that I have read this submission and believe that I have an appropriate level of expertise to confirm that it is of an acceptable scientific standard.

# Peer review: Reviewer response for version 2

**DOI:** 10.5256/f1000research.140907.r156632

**Published:** 2023-01-03

**Authors:** Timothy M. Errington

**Affiliations:** 1 Centre for Open Science, Charlottesville, VA, USA

I thank the authors for adding additional clarity and context to their protocol. This is a well thought out study, with appropriate context and description of limitations. I look forward to the outcomes of the experiments.

Is the study design appropriate for the research question?

Yes

Are the 3Rs implications of the work described accurately?

Yes

Have the authors pre-specified sufficient outcome-neutral tests for ensuring that the results obtained can test the stated hypotheses, including positive controls and quality checks?

Yes

Is the rationale for, and objectives of, the study clearly described?

Yes

Are sufficient details of the methods provided to allow replication by others?

Yes

Are the datasets clearly presented in a useable and accessible format?

Yes

Are a suitable application and appropriate end-users identified?

Yes

Reviewer Expertise:

I have expertise in replicability, reproducibility, metascience, cellular and biochemical assays (e.g., PCR, imaging), and some animal protocols (e.g., tail-vein injections, xerograph models). I do not have expertise in rat tickling (or rat handling specifically) nor expertise in behavioural test, such as EPM and OF described in this Stage 1 Registered Report.

I confirm that I have read this submission and believe that I have an appropriate level of expertise to confirm that it is of an acceptable scientific standard.

# Peer review: Reviewer response for version 1

**DOI:** 10.5256/f1000research.137980.r153996

**Published:** 2022-10-24

**Authors:** Theresa M. Kisko

**Affiliations:** 1 Social and Affective Neuroscience – Brain and Cognition Research Unit, KU Leuven, Leuven, Belgium

Summary: The goal of this protocol is to evaluate the standard “Panksepp” tickling protocol against the authors' own playful handling (PH) protocol and assess which is a more ideal way to ensure the maximum positive experiences for the animals. This is done by varying the amount of pinning and providing a more interactive/reciprocal type of heterospecific play. The authors hypothesise that less pinning and more “playful interactions” will create an overall more positive experience – assessed through primary response variables Total 50-kHz USV and variability of 50-kHz USV across treatments. The second objective is to assess the physiological and behavioural effects of the various pinning and playful handling protocols. The hypothesis is that less pinning and more PH will create less variable behavioural and physiological responses. The authors will assess this through an open field, elevated plus maze and post-mortem tissue measures (pro-inflammatory cytokines) and faecal corticosterone.

 Evaluation: The rationale for refining the tickling protocol is justly described and the authors provide compelling evidence from their previous studies, with descriptions and example figures and graphs, that their playful handling protocol is efficient and that it reduces the variability across individuals. Their methods suggest that more rats are finding the playful handling experience and interaction positive.

 I quite like how the authors have considered the reciprocal nature of playful interactions and incorporated this into their playful handling protocol as the standard tickling does not take this into account enough. For play interactions to remain playful, there needs to be a certain amount of reciprocity and I think the way that the authors can assess the individual rats’ responses to the experimenter hand and interact with the rat in a more dynamic way is an excellent way to ensure that the result is a positive interaction rather than a forced USV release that may not actually be enjoyable. Naturally creating a more fluid emission of 50-kHz USV in contrast to high rates of pinning and a more “coerced” 50-kHz USV emission can provide less confounds in experimental testing and ensure a more positive welfare condition. When Panksepp created and published the standard tickling protocol the field of affective USV research I would argue was still in its infancy. However, over the last two decades, we have learned much more and, therefore, I quite agree that the “older” standardized protocols should be re-evaluated and updated to reflect the knowledge we now know, especially for the benefit of the animals in our care.

 The experimental methods outlined for objectives 1 and 2 are straightforward and well thought out. I have only a couple of minor queries for them:

1. I would question the use of Audacity software in USV analysis though, as in my experience with the USV analysis it is not a common software for assessment. I have never heard of this software being used for USVs. There are some that are more specifically targeted for ultrasonic vocalizations and might possibly provide a more accurate assessment, especially when it comes to the categorization of 50-kHz USV subtypes. There are even several freeware versions that can be used that are user-friendly and intuitive (i.e. Deepsqueek). Have the authors considered comparing one or two files assessed with Audacity to one or two assessed with a specific vocalization software typically used in USV research, i.e. Avisoft or Deepsqueek?
2. In terms of the secondary response variable (a) in objective 1, what is the question you are exploring with subtypes? Essentially, I would ask why are you wanting to look at subtypes? Are you looking to see if certain subtypes reflect a more positive interaction? For example, would one expect that with less pinning the rats emit less trills or frequency-modulated subtypes, or just the variability of subtypes emitted during tickling? Are you planning to coordinate the subtype emissions with the behaviour occurring, i.e. while chasing the experimenter's hand, what calls do they primarily emit? This can be quite a complicated assessment and requires accurate synchronization of behaviour and USV times.
3. How do you plan to do the categorization? By an independent observer manually categorizing or do you plan to use software that is trained for automated categorizations? There are obviously pros and cons to both, but I think this is an important point to consider when planning categorical USV analysis.

In terms of general experimental overview, controls are provided for each instance and the experimental treatments are balanced and pseudorandomized between groups of animals. The collection of tissue and faecal samples to assess physiological responses as well as using standard behavioural assays to further evaluate the effects or consequences of the playful handling protocols provides a nice balance to purely behavioural and vocalization data. This assessment could strengthen results and provide compelling evidence for an effective protocol.

 Diagrams and figures in the protocol are well described and include ample details to allow for sufficient replication studies.  All the statistical analyses are well outlined and have been justly described based on the variables and research questions.

 Study limitations are outlined and clearly have been thought out.

 In general, I quite like the protocol refinement and I agree that it is justified, and the authors provide compelling reasons and evidence to support their hypothesis. I look forward to the outcome of the experiments.

Is the study design appropriate for the research question?

Yes

Are the 3Rs implications of the work described accurately?

Yes

Have the authors pre-specified sufficient outcome-neutral tests for ensuring that the results obtained can test the stated hypotheses, including positive controls and quality checks?

Yes

Is the rationale for, and objectives of, the study clearly described?

Yes

Are sufficient details of the methods provided to allow replication by others?

Yes

Are the datasets clearly presented in a useable and accessible format?

Yes

Are a suitable application and appropriate end-users identified?

Yes

Reviewer Expertise:

Social play behaviour in rats, Ultrasonic vocalizations, Social and affective behaviours and communication.

I confirm that I have read this submission and believe that I have an appropriate level of expertise to confirm that it is of an acceptable scientific standard.

# Response

**Published:** 2022-11-15

**Authors:** Vincent Bombail

**Affiliations:** SRUC, UK

***We wish to thank this reviewer for their encouragements and positive feedback. Some good points are raised, we have addressed the questions below.***

 I would question the use of Audacity software in USV analysis though, as in my experience with the USV analysis it is not a common software for assessment. I have never heard of this software being used for USVs. There are some that are more specifically targeted for ultrasonic vocalizations and might possibly provide a more accurate assessment, especially when it comes to the categorization of 50-kHz USV subtypes. There are even several freeware versions that can be used that are user-friendly and intuitive (i.e. Deepsqueek). Have the authors considered comparing one or two files assessed with Audacity to one or two assessed with a specific vocalization software typically used in USV research, i.e. Avisoft or Deepsqueek?

***This is a fair point, we have previously used Audacity for visual detection of USV, but we will hopefully gain enough expertise in the use of Deepsqueak, to compare both detection methods.***

 In terms of the secondary response variable (a) in objective 1, what is the question you are exploring with subtypes? Essentially, I would ask why are you wanting to look at subtypes? Are you looking to see if certain subtypes reflect a more positive interaction? For example, would one expect that with less pinning the rats emit less trills or frequency-modulated subtypes, or just the variability of subtypes emitted during tickling? Are you planning to coordinate the subtype emissions with the behaviour occurring, i.e. while chasing the experimenter's hand, what calls do they primarily emit? This can be quite a complicated assessment and requires accurate synchronization of behaviour and USV times[SB1] .

***We have added a sentence in Data collection/Confirmatory hypothesis, to clarify this use of USV subtypes (‘We will investigate whether certain USV types might be preferably associated with certain treatments’).***

 How do you plan to do the categorization? By an independent observer manually categorizing or do you plan to use software that is trained for automated categorizations[SB2] ? There are obviously pros and cons to both, but I think this is an important point to consider when planning categorical USV analysis.

***We aim to categorise our USVs according to the previously published description (Wright et al., 2010), as we feel this common referential will contributes toward an improved understanding of rat USV production.***

# Peer review: Reviewer response for version 1

**DOI:** 10.5256/f1000research.137980.r150622

**Published:** 2022-10-12

**Authors:** Timothy M. Errington

**Affiliations:** 1 Centre for Open Science, Charlottesville, VA, USA

The Stage 1 Registered Report outlines the rationale, study design, hypotheses, and analysis strategy for testing whether updated rat tickling protocol (playful handling: PH) leads to a higher and less variable induction of ultrasonic vocalisation (USV) compared to the standard tickling protocol, indictive of an increased positive playful interaction. The authors also describe exploratory experimentation to examine whether the difference in tickling protocols leads to less variation in behavioural and physiological tests

 Overall, this is a well thought out and described protocol. Suggestions below are considerations for the authors to increase transparency of the Stage 1 Registered Report and experimental factors to consider in their design or to include as limitations, or discussion.

1. In Hypotheses section, suggest listing Hypothesis 1 as Confirmatory Hypothesis 1a and Confirmatory Hypothesis 1b (or just making them 1 and 2?). And Hypothesis 2 as Exploratory Hypothesis 1. That way it is even clearer to readers what is confirmatory vs exploratory.
2. The authors should include a brief statement of why EMP and OF are the tests they are using.
3. How many pins are normally done in the standard tickling procedure? Is P4 equivalent to it? It sounds like there is quite a bit of variation in how the standard tickling procedure is done including number per treatment and number of treatments. On number of treatments, the authors will record and analysis after just 1 treatment (Phase 1) and not also at Phase 2. Since the data are recorded it seems useful to analysis at both points (possibly even incorporating it in the statistical design?)
4. Related to point 3, what is the need for a gap between Phase 1 and Phase 2? That is, why not just move from the treatment on days 9-11 into the EPM and OF testing? Is it because the rats should have two treatment sessions spaced apart by 5 days before the behavioural and physiological tests? Either way can the authors elaborate this design decision.
5. Maybe I missed it, but will the cages assigned to one treatment (e.g., H0) in Phase 1 also be in the same treatment in Phase 2 or will they be randomized?
6. It is nice to see the pseudo-randomization. I was going to recommend also introducing the randomization for the order of cages treated (in addition to the order of rats tested in each cage), but it looks like this is part of the design. However, it is only stated once in Objective 1 experimental design. I suggest moving this up earlier in the protocol and possibly repeating it where appropriate, such as when discussing the pseudo-randomization design and rat ordering as it was unclear until this single sentence.
7. Related to the need to randomize where possible, especially since the rat handler will not be blind to treatments, I think an unfortunate weakness of this protocol, although I understand why from a practical sense, is the lack of variability of researcher. That is, a single researcher at a single institution with a single rat strain is being used. Three related thoughts on this:
  a) The authors might consider discussing the impact of features that are not being accounted for that might impact the outcome (e.g., experience of the handler performing the standard tickling protocol, experience of the handler performing the PH protocol, sex of the researcher (e.g., Sorge *et al*., 2014 ^1^).
  b) I am not an expert in this technique, but having a strong proponent of the standard rat tickling protocol could likely provide some quite useful critique – essentially an adversarial collaboration that help with pre-commitment (Brian Nosek and I wrote a piece about it here: https://www.nature.com/articles/d41586-020-02142-6 - and note, I’m not suggesting you cite this, only providing for background with the concept if helpful).
  c) A consideration to keep in mind for the discussion of the outcomes of this (yes, getting ahead for a moment), is one way to address this would be through a ‘many-labs’ style replication of these findings, essentially introducing the variation of researcher, strain, laboratory, etc. Again, this study would need to be completed with results to warrant a ‘many-labs’ approach, but sharing now for future consideration.
8. Another potential factor to consider is time of day of the treatment. It is unclear if that is held constant. That is, will the treatment always occur at the same time of day relative to the 12h:12h light cycle of the rats or if it will also be variable across cohorts? I am unsure if this could have an impact on behaviour, but raising as something potentially to track and share at the completion of the study.
9. For forming cage pairs for each cohort, while you will always achieve 4 cages of female and 4 cages of male (by design), it seems it might not always be possible to form pairs based on provenance since that will depend on litter size. It is less of a concern if there are rats from many litters, but is there a chance of receiving more than 4 rats of one sex from the same litter during any of the cohorts? It sounds like the authors have already discussed this with the supplier of the rats, but it’s unclear how this is taken into account in the randomization strategy if this issue arises.
10. Is there any consideration of whether a rat is unable to continue? For example, if a rat forms an infection during the habitation caused by something else, will the authors exclude the rat from the experiment? If any of this is known a priori it would be good to mention, if not, then transparency during report will be sufficient.
11. I’d recommend adding RRIDs where appropriate (e.g., rat strain).
12. It sounds like for the secondary variables of interest (e.g., when coding the anticipatory play behaviour), these will be coded by a researcher from the video recordings and that the researchers will be blinded to the behavioural and physiological tests. This is again great to see, however if the authors could share a little more detail of how they will do this, that would be helpful for transparency.
13. For analyses, the authors should include more transparency and more explicitness for confirmatory tests. For example:
  a) The *p*-value threshold (assuming 0.05 based on power analysis) they will consider as significant.
  b)The comparisons they will perform, which is especially important for the primary response variables. So for example, is the test for total 50 kHz USV a contrast of P0 and P1 compared to P4 and Control? Or are the authors suggesting multiple contrasts (e.g., P0 vs P4, P1 vs P4, etc). For example, the legend for Figure 3ii suggests it would be P0 and P1 vs P4 (excluding control).
  c) If relevant, how are multiple tests being corrected for?
  d) What approaches will be used to test data fit the assumptions of the tests (e.g., what specific tests for normality), and same with data transformations (e.g., what are viable options to consider and what is the workflow to consider it sufficient) – this can help make the decision tree the authors will use on whether to use one test vs an alternative more transparent *a priori*.
  e) And while not necessarily needed now, it would be beneficial to know the authors plan to report effect sizes and uncertainty in their analyses.

Is the study design appropriate for the research question?

Yes

Are the 3Rs implications of the work described accurately?

Yes

Have the authors pre-specified sufficient outcome-neutral tests for ensuring that the results obtained can test the stated hypotheses, including positive controls and quality checks?

Yes

Is the rationale for, and objectives of, the study clearly described?

Yes

Are sufficient details of the methods provided to allow replication by others?

Yes

Are the datasets clearly presented in a useable and accessible format?

Yes

Are a suitable application and appropriate end-users identified?

Yes

Reviewer Expertise:

I have expertise in replicability, reproducibility, metascience, cellular and biochemical assays (e.g., PCR, imaging), and some animal protocols (e.g., tail-vein injections, xerograph models). I do not have expertise in rat tickling (or rat handling specifically) nor expertise in behavioural test, such as EPM and OF described in this Stage 1 Registered Report.

I confirm that I have read this submission and believe that I have an appropriate level of expertise to confirm that it is of an acceptable scientific standard, however I have significant reservations, as outlined above.

## References

[1] : Olfactory exposure to males, including men, causes stress and related analgesia in rodents. *Nat Methods* .2014;11(6) : 10.1038/nmeth.2935 629-32 10.1038/nmeth.2935 24776635

# Response

**Published:** 2022-11-15

**Authors:** Vincent Bombail

**Affiliations:** SRUC, UK

***We wish to thank this reviewer for their thorough and constructive criticism, and we hope our attempt at addressing their questions has brought more clarity to our manuscript.***

 The Stage 1 Registered Report outlines the rationale, study design, hypotheses, and analysis strategy for testing whether updated rat tickling protocol (playful handling: PH) leads to a higher and less variable induction of ultrasonic vocalisation (USV) compared to the standard tickling protocol, indictive of an increased positive playful interaction. The authors also describe exploratory experimentation to examine whether the difference in tickling protocols leads to less variation in behavioural and physiological tests

 Overall, this is a well thought out and described protocol. Suggestions below are considerations for the authors to increase transparency of the Stage 1 Registered Report and experimental factors to consider in their design or to include as limitations, or discussion.

 In Hypotheses section, suggest listing Hypothesis 1 as Confirmatory Hypothesis 1a and Confirmatory Hypothesis 1b (or just making them 1 and 2?). And Hypothesis 2 as Exploratory Hypothesis 1. That way it is even clearer to readers what is confirmatory vs exploratory.

***This is a good idea and we have updated the text to follow this advice.***

 The authors should include a brief statement of why EMP and OF are the tests they are using.

***We have added explanatory sentences in the methods section: ‘The EPM and OF tests consist in quantifying exploration behaviour in 2 different structures, that both consist in an area with open spaces (the centre of the OF and the open arms of the EPM) and an area with relatively more enclosed spaces (the periphery of the OF and the closed arms of the EPM).  For rats, this ambiguity creates a conflict between the thigmotaxis (preference for proximity to walls) and the motivation to explore open spaces, which is used to assess the emotional response to those novel experiences, and to test for anxiety levels.’***

 How many pins are normally done in the standard tickling procedure? Is P4 equivalent to it? It sounds like there is quite a bit of variation in how the standard tickling procedure is done including number per treatment and number of treatments. On number of treatments, the authors will record and analysis after just 1 treatment (Phase 1) and not also at Phase 2. Since the data are recorded it seems useful to analysis at both points (possibly even incorporating it in the statistical design?)

***The protocol from Cloutier et al. (2018) describes there should be 4 to 5 pins per 15 s (2-4s per pin). We also postulated a 30 s treatment would be a practical and effective duration to achieve induction of most individual rat positive affect (based on LaFollette et al., 2018, and our own findings). Our protocol and treatment design synthesise these requirements.***

 Related to point 3, what is the need for a gap between Phase 1 and Phase 2? That is, why not just move from the treatment on days 9-11 into the EPM and OF testing? Is it because the rats should have two treatment sessions spaced apart by 5 days before the behavioural and physiological tests? Either way can the authors elaborate this design decision.

***There is no gap between Phases 1 and 2, phase 2 starts the next day. To clarify we have added the word ‘next’, in experimental design for objective 2.***

 Maybe I missed it, but will the cages assigned to one treatment (e.g., H0) in Phase 1 also be in the same treatment in Phase 2 or will they be randomized?

***For phase 2, to investigate the behavioural and physiological correlates of positive experiences, the animals and their treatments will be the same as in phase 1.***

 It is nice to see the pseudo-randomization. I was going to recommend also introducing the randomization for the order of cages treated (in addition to the order of rats tested in each cage), but it looks like this is part of the design. However, it is only stated once in Objective 1 experimental design. I suggest moving this up earlier in the protocol and possibly repeating it where appropriate, such as when discussing the pseudo-randomization design and rat ordering as it was unclear until this single sentence.

***We have added this to the testing in objective 2: ‘their order of passage will be pseudo-randomised’.***

 Related to the need to randomize where possible, especially since the rat handler will not be blind to treatments, I think an unfortunate weakness of this protocol, although I understand why from a practical sense, is the lack of variability of researcher. That is, a single researcher at a single institution with a single rat strain is being used. Three related thoughts on this:

 a) The authors might consider discussing the impact of features that are not being accounted for that might impact the outcome (e.g., experience of the handler performing the standard tickling protocol, experience of the handler performing the PH protocol, sex of the researcher (e.g., Sorge et al., 20141).

***The inclusion of further experimenters in the design could constitute another source of variation, but we agree the involvement of a single experienced male experimenter might constitute a limitation. Over time there will be other sources of variation (e.g. dietary molecules excreted through sweat, fluctuation in mood and playfulness), but the study of human-animal interactions requires such human intervention. We have to start somewhere, and although this will not the ultimate testing of the hypothesis, this will be a first attempt that might next be transposed to other laboratories and rat strains. We have added these points in the study limitations section.***

 b) I am not an expert in this technique, but having a strong proponent of the standard rat tickling protocol could likely provide some quite useful critique – essentially an adversarial collaboration that help with pre-commitment (Brian Nosek and I wrote a piece about it here: https://www.nature.com/articles/d41586-020-02142-6 - and note, I’m not suggesting you cite this, only providing for background with the concept if helpful).

***Thank you for this interesting read. This protocol, and the study in general, were designed inclusive of different perspectives on tickling, and in collaboration with our co-author Megan LaFollette, who is a proponent of the standard protocol (i.e. the Panksepp protocol with pinning described in Cloutier et al., 2018).***

 c) A consideration to keep in mind for the discussion of the outcomes of this (yes, getting ahead for a moment), is one way to address this would be through a ‘many-labs’ style replication of these findings, essentially introducing the variation of researcher, strain, laboratory, etc. Again, this study would need to be completed with results to warrant a ‘many-labs’ approach, but sharing now for future consideration.

***This is very interesting. This has been incorporated in the response to point a) above, in the study limitations sections***

 Another potential factor to consider is time of day of the treatment. It is unclear if that is held constant. That is, will the treatment always occur at the same time of day relative to the 12h:12h light cycle of the rats or if it will also be variable across cohorts? I am unsure if this could have an impact on behaviour, but raising as something potentially to track and share at the completion of the study.

***We have specified time of treatments in the ‘Acclimation, treatment allocation, housing and husbandry section’.***

 For forming cage pairs for each cohort, while you will always achieve 4 cages of female and 4 cages of male (by design), it seems it might not always be possible to form pairs based on provenance since that will depend on litter size. It is less of a concern if there are rats from many litters, but is there a chance of receiving more than 4 rats of one sex from the same litter during any of the cohorts? It sounds like the authors have already discussed this with the supplier of the rats, but it’s unclear how this is taken into account in the randomization strategy if this issue arises.

***We have discussed this with the supplier (CRL) and we will not receive more than 4 rats of one sex from the same litter.***

 Is there any consideration of whether a rat is unable to continue? For example, if a rat forms an infection during the habitation caused by something else, will the authors exclude the rat from the experiment? If any of this is known a priori it would be good to mention, if not, then transparency during report will be sufficient.

***We have updated the text to include this information. We will follow local guidelines should rat injury occur. If this were to happen, this information would be described in the methods section, for full transparency about the fate of our experimental rats.***

 I’d recommend adding RRIDs where appropriate (e.g., rat strain).

***We have added the following information to the materials section: Research Resource Identification, RRID:RGD_2308816***

 It sounds like for the secondary variables of interest (e.g., when coding the anticipatory play behaviour), these will be coded by a researcher from the video recordings and that the researchers will be blinded to the behavioural and physiological tests. This is again great to see, however if the authors could share a little more detail of how they will do this, that would be helpful for transparency.

***It will be impossible to deduce the nature of the treatment, since all treatment orders will have been pseudo randomised, and none of the experimenters involved has a good enough memory to remember the treatment group of 64 different cages.***

 For analyses, the authors should include more transparency and more explicitness for confirmatory tests. For example:

 a) The p-value threshold (assuming 0.05 based on power analysis) they will consider as significant.

***This has now been specified in the manuscript.***

 b)The comparisons they will perform, which is especially important for the primary response variables. So for example, is the test for total 50 kHz USV a contrast of P0 and P1 compared to P4 and Control? Or are the authors suggesting multiple contrasts (e.g., P0 vs P4, P1 vs P4, etc). For example, the legend for Figure 3ii suggests it would be P0 and P1 vs P4 (excluding control).

***All treatments will be compared***

 c) If relevant, how are multiple tests being corrected for?

***We have amended the text accordingly: ‘To account for potential type I errors that may arise from multiple comparisons, we will use Bonferroni correction or equivalent (depending on data distribution)’.***

 d) What approaches will be used to test data fit the assumptions of the tests (e.g., what specific tests for normality), and same with data transformations (e.g., what are viable options to consider and what is the workflow to consider it sufficient) – this can help make the decision tree the authors will use on whether to use one test vs an alternative more transparent a priori.

***We will use classical tests such as the Bartlett's test for homogeneity of variances and Anderson-Darling test for normality. If necessary, data might be transformed (e.g. square root transformation)***

 e) And while not necessarily needed now, it would be beneficial to know the authors plan to report effect sizes and uncertainty in their analyses.

***We have added : ‘In the spirit of transparency, we will report effect sizes and uncertainty in our analyses.’***
